# An iliac arteriovenous fistula associated with leg swelling 2 years after the removal of a hemodialysis catheter: A case report

**DOI:** 10.1097/MD.0000000000041281

**Published:** 2025-01-17

**Authors:** Tao Du, Xing Zheng

**Affiliations:** a The Third Hospital of Mianyang, Sichuan Mental Health Center, Mianyang, Sichuan, China.

**Keywords:** arteriovenous fistula, case report, catheter, complication, dialysis

## Abstract

**Rationale:**

Temporary central venous catheters are commonly used for patients who require emergency hemodialysis. In rare instances, this procedure can result in a very serious complication known as arteriovenous fistula (AVF). Although some cases of femoral arteriovenous fistula have been reported previously in the literature; however, the incidence of iliac AVF precipitated by a hemodialysis catheter is exceedingly uncommon. The purpose of this study is to report a case in which a hemodialysis catheter caused a femoral arteriovenous fistula and to describe the successful treatment, thereby providing a reference for the diagnosis and treatment of such conditions in the future.

**Patient concerns:**

Herein, we report the case of a patient with diabetes and end-stage kidney disease (ESKD) who presented with an iliac arteriovenous fistula as a complication of hemodialysis catheter insertion into the right femoral vein. Two years after hemodialysis catheter removal, the patient was admitted to our institution with swelling of the right lower limb and calf pain.

**Diagnosis:**

The patient was subsequently diagnosed with an iliac arteriovenous fistula by computed tomography angiography and angiography.

**Interventions:**

The patient was transferred to the Vascular Surgery Department for surgical treatment.

**Outcomes:**

The patient’s leg swelling improved after surgery. After 1 year of follow-up, there was no occurrence of swelling in the patient’s right leg.

**Lessons:**

If the patient has a history of femoral venous catheter placement and develops limb swelling, in addition to considering the presence of a femoral arteriovenous fistula, it is important to consider the possibility of an iliac AVF. Iliac AVF are rare disorders. The primary goal for treatment of IAVF is to relieve associated symptoms. Based on the results of this study, the endovascular approach is safe and effective for the treatment of iliac AVF. Iliac AVFs are rare disorders. The primary goal of treatment for iliac AVFs is to relieve associated symptoms. Based on the results of this study, the endovascular approach is safe and effective in the treatment of iliac AVFs.

## 1. Introduction

Native arteriovenous fistulas (AVFs) are the preferred form of first-line vascular access for hemodialysis (HD) in patients with end-stage renal disease (ESKD). However, timely AVF creation is not possible in every patient for a variety of reasons, including delayed presentation and requirement for emergency HD. Temporary hemodialysis catheterization is commonly used for vascular access in such situations, although this practice can lead to certain complications, including bleeding, infection, hematoma and deep venous thrombosis. The formation of an iatrogenic fistula between an artery and its adjacent vein as a consequence of temporary hemodialysis catheter placement is a rare complication. Some cases involving femoral arteriovenous fistulas have been reported in the literature; however, cases of iliac arteriovenous fistulas are rarely reported. Herein, we report the case of a diabetic patient with end-stage kidney disease (ESKD) who presented with an iliac arteriovenous fistula as a complication following the insertion of a temporary hemodialysis catheter into the right femoral vein.

## 2. Case presentation

**Figure 1.**
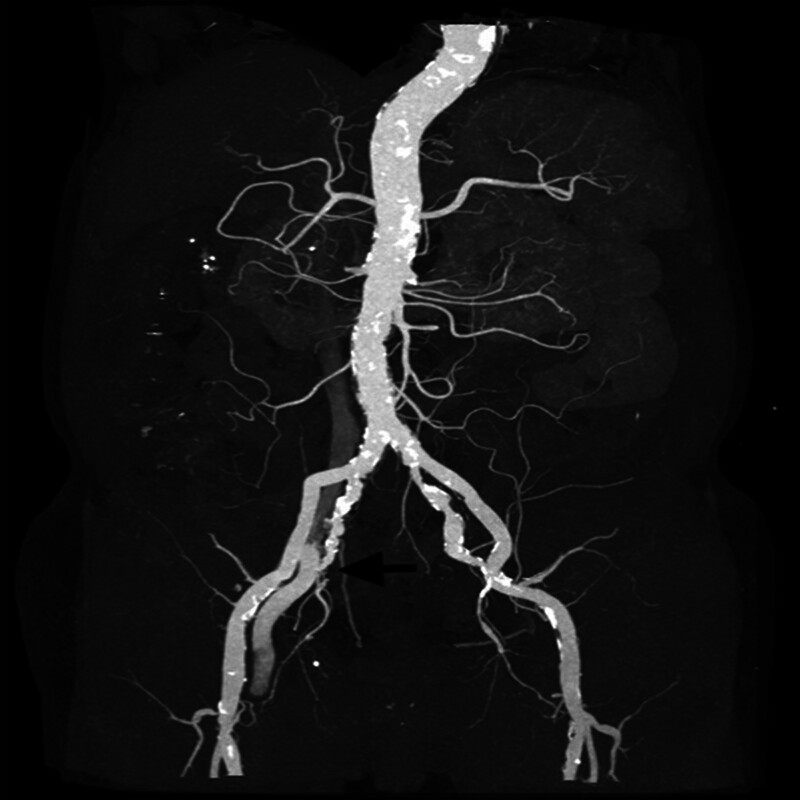
CTA revealing the arteriovenous communication between the right internal iliac artery and the right common iliac vein. CTA = computed tomography angiography.

**Figure 2.**
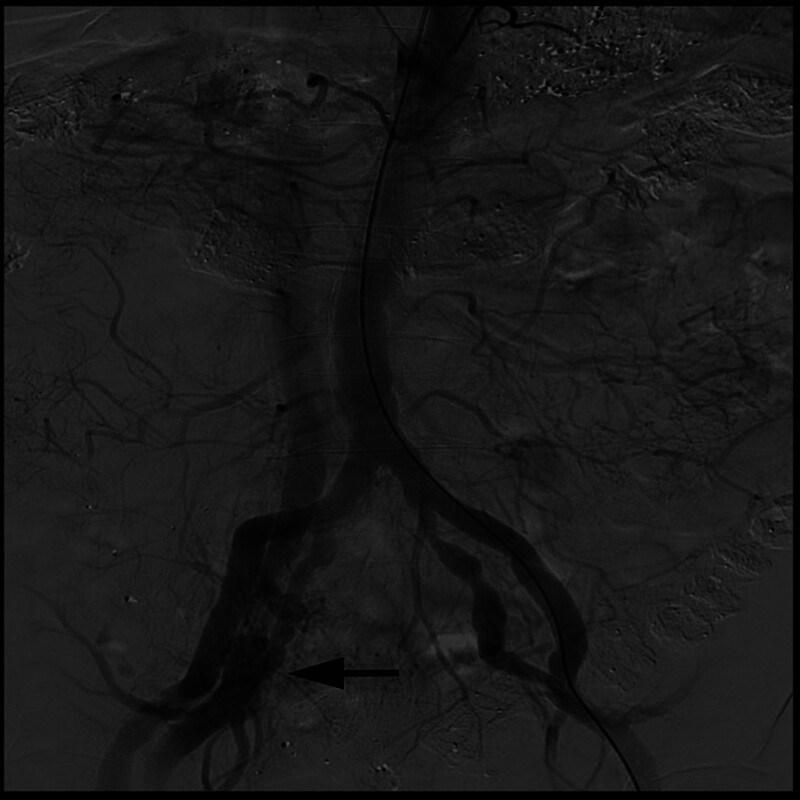
Angiography revealing the arteriovenous communication between the right internal iliac artery and the right common iliac vein.

A 71-year-old male patient was diagnosed with ESKD due to diabetic nephropathy and a left brachiocephalic AVF was created. Two weeks later, the patient underwent HD via a right femoral vein temporary vascular catheter (double-lumen, 11.5Fr, China) which was inserted when he presented with symptoms of fluid overload. Notably, ultrasonography was not performed during the catheter insertion. Once the AVF had matured, the right femoral catheter was removed after being in place for 1 month. Two years after catheter removal, the patient was admitted to our institution with swelling of the right lower limb and calf pain. Color Doppler Ultrasound detected an AVF between the right internal iliac artery and the right common iliac vein, which was subsequently confirmed by computed tomography angiography and angiography (Figs. 1 and 2). Subsequently, the patient was transferred to the Vascular Surgery Department for surgical treatment. During surgery, abdominal aortography revealed smooth walls of the bilateral common iliac arteries and external iliac arteries and imaging of the right common iliac vein. Angiography revealed 2 branches of the middle and distal internal iliac arteries communicating with the right common iliac vein. Right femoral vein angiography revealed a 95% stenosis of the right common iliac vein. This was treated by dilation of the stenotic lesion immediately beneath the bifurcation of the right common iliac artery using a balloon and stent insertion (Fluency, 14 mm × 60 mm, FVL14060) and coil embolization (Boston, 9 mm × 2.7 mm, M001372960) of the right internal iliac artery. Repeat angiography revealed that the fistula had been successfully repaired. The patient’s leg swelling improved and he was discharged 5 days after surgery. After 1 year of follow-up, there was no occurrence of swelling in the patient’s right leg. Approximately 18 months subsequent to the initial diagnosis, the patient succumbed to complications arising from the novel coronavirus infection.

## 3. Discussion

Some papers have reported arteriovenous fistula as a complication of hemodialysis catheter insertion into the right internal jugular vein.^[1–5]^ The most common complications in this practice are leg swelling and calf pain;^[6–8]^ however the time of onset of these symptoms remains uncertain. In a previous report, Gunawarden et al^[7]^ described a case involving a right superficial femoral AVF with severe leg swelling after 2 weeks. In another report, Jin et al^[8]^ reported a case of AVF-associated leg swelling 6 months after the removal of a hemodialysis catheter. However, cases of iliac arteriovenous fistulas are very rare. In this case, an iliac arteriovenous fistula developed as a complication following the insertion of a temporary hemodialysis catheter into the right femoral vein. The patient’s first symptom was limb swelling, which occurred 2 years after the hemodialysis catheter was removed. In our patient, it is likely that the right internal iliac artery was damaged during catheter insertion into the right femoral vein. It is likely that removal of the catheter made the channel between the vessels patent, thus leading to progressive limb swelling. However, leaving the catheter for an extended period may damage the vessel wall. It is possible that the tip of the catheter may have eroded the vessel wall, resulting in endothelial damage. A previous study reported a case of persistent left-sided superior vena cava with potential central venous catheter vascular erosion following insertion of a left-sided internal jugular dialysis catheter.^[9]^

Femoral arteriovenous fistulas are more common than iliac arteriovenous fistulas; this is because the femoral artery is closer to the puncture point and is more likely to incur damage. In particular, real-time ultrasound guidance is not normally used during catheter insertion. If left untreated, a fistula can lead to high-output heart failure, arrhythmias, or thromboembolic episodes. If the patient has a history of femoral venous catheter placement and develops limb swelling, in addition to considering the presence of a femoral arteriovenous fistula, it is important to consider the possibility of an iliac arteriovenous fistula. Color Flow Duplex Ultrasonography is a reliable and simple examination for this relatively rare condition. Subsequently, angiography can be used to confirm the diagnosis.

Currently, there is no consensus regarding the optimal therapeutic strategy for iliac AVFs. Due to the potential for serious complications, conservative treatment with compression therapy is usually not suitable for treating iliac AVF. Furthermore, open repair may result in greater surgical trauma and lead to more complications.^[10]^ Given the patient’s advanced age, chronic kidney failure requiring maintenance dialysis, and the presence of obvious edema and pain in the right lower limb, we opted for endovascular treatment.

This case study has several limitations in explaining this complication: Due to the patient dying from other diseases, the follow-up period was short. During the 1-year follow-up period, the patient’s symptoms did not recur, but no imaging data were available to further evaluate the patient’s iliac vessel condition.

## 4. Conclusion

Iliac AVF is a rare complication that can occasionally be fatal. If there a suspicion of AVF, appropriate tests such as duplex scans and angiography are highly indicated. The incidence of iliac AVF precipitated by hemodialysis catheter is exceedingly uncommon. The primary goal for treatment of iliac AVF is to relieve associated symptoms. Based on the results of this study, the endovascular approach is safe and effective for the treatment of iliac AVF.

## Author contributions

**Data curation:** Xing Zheng.

**Formal analysis:** Xing Zheng.

**Writing – original draft:** Tao Du.

**Writing – review & editing:** Tao Du.
